# Journal peer review: a bar or bridge? An analysis of a paper’s revision history and turnaround time, and the effect on citation

**DOI:** 10.1007/s11192-017-2630-5

**Published:** 2018-01-03

**Authors:** J. Rigby, D. Cox, K. Julian

**Affiliations:** Manchester Institute of Innovation Research, Alliance Manchester Business School, The University of Manchester, c/o Room 8.23, Harold Hankins Building, Oxford Road, Manchester, M13 9PL UK

**Keywords:** Turnaround time, Peer review, Journal, Author

## Abstract

Journal peer review lies at the heart of academic quality control. This article explores the journal peer review process and seeks to examine how the reviewing process might itself contribute to papers, leading them to be more highly cited and to achieve greater recognition. Our work builds on previous observations and views expressed in the literature about (a) the role of actors involved in the research and publication process that suggest that peer review is inherent in the research process and (b) on the contribution reviewers themselves might make to the content and increased citation of papers. Using data from the journal peer review process of a single journal in the Social Sciences field (Business, Management and Accounting), we examine the effects of peer review on papers submitted to that journal including the effect upon citation, a novel step in the study of the outcome of peer review. Our detailed analysis suggests, contrary to initial assumptions, that it is not the time taken to revise papers but the actual number of revisions that leads to greater recognition for papers in terms of citation impact. Our study provides evidence, albeit limited to the case of a single journal, that the peer review process may constitute a form of knowledge production and is not the simple correction of errors contained in submitted papers.

## Introduction

This article explores journal peer review and seeks to examine how the reviewing process might itself contribute to the citedness of papers. Our paper employs a range of bibliometric data, including that on the time taken by the journal from the moment a paper is received to the point in time when the journal decides finally to accept or reject the paper, the count of submissions of papers, and initial editorial decisions about a paper on receipt by a journal. We relate these measures to the citedness of the published papers. Our work builds on previous observations about (a) the role of actors involved in the research and publication process which has argued that peer review is inherent in the research process (Rigby and Edler 2005); (b) work which emphasises the contribution which reviewers themselves make to the content and therefore citedness of papers, particularly in the social and behavioural sciences; and (c) the claims by Cowley (2015) of peer review as systemic, distributed, complex knowledge production process within which larger frame of reference journal peer review occurs. In respect of these observations, and in particular our focus on the role of reviewers, we note the words of Bakanic et al. (1987, p. 641) who claimed that ‘expert peer participation may not come until after the journal’s review is underway… and where manuscript review, revision, and resubmission process are vital contributions to the construction of the scholarship reported’—a claim made fully a decade and a half before Frey (2003) asked authors to choose between writing their own work and academic success.

## Literature

Peer review is a decision making activity playing a central role in scientific research with analogues in many social processes including politics where debates over direct and representative democracy (see Cartledge (2016) for a recent discussion) carry echoes of the controversies and developments in scientific practice. In science, peer review’s central objective is to ensure that, as far as possible, knowledge claims are reasonable in that they do not overstep the bounds of what can logically or empirically be claimed (Hemlin and Rasmussen 2006). Writing nearly 30 years ago now, Chubin and Hackett (1990) made the important distinction between two main contexts in which peer review operates, that of *grant* or *proposal review*, where research is yet to be done, and *journal peer review*, where the research has been done and knowledge claims are being put forward. While peer review is a vital feature at the point of resource allocation (i.e. grant winning) see for example (Wessely and Wood 1999), and by some considered to be more important in that it can lead directly to the provision of resources for research, it is journal peer review that is most often thought of as the quintessence of academic quality control. So central is peer review to the production of academic knowledge that peer review has itself very been heavily subject to evaluation and investigation. However, as Chubin and Hackett (1990, p. 96) have observed: ‘… like proposal review, the journal peer review system is difficult to study, both because it is inherently hard to observe and because its members actively resist investigation.’ Their classic study of peer review (p. 98) goes on to note the challenges for such a research agenda: ‘These events (the harassment and of Douglas Peters and Stephen Ceci) [they say] bring to mind J.R.R. Tolkien’s warning: “Do not meddle in the affairs of wizards as they are subtle and quick to anger”. So too are journal editors (and their sponsors, publishers and professional societies), and for that reason the ambitious agenda of necessary research about journal peer review may remain largely unexplored.’

Our purpose in this paper is to try to understand better what is happening in the peer review process and in particular to examine those features of peer review that might give insight into how the impact of a paper is affected by the way it is reviewed, and how much effort is spent on revising it, both from the input of the authors and from the side of the journal editor and referees. We observe that previous studies have indeed considered the reviewing process as more than simple a judgemental activity which reaches yes/no; go/no go decisions but one which has an influence upon the quality of the published article. And while such work has examined critically the features of the process that affect quality, the studies we have examined have not specifically related key aspects of the reviewing of a paper to its subsequent citation. Our study is a small empirical one in which we use a data set that explores our claim of a possible link between the peer reviewing process and the ensuing interest in the paper. Our analysis allows us to draw some tentative conclusions and we then consider the implications for the future study of peer review.

Studies of peer review are diverse in terms of methods adopted and in terms of the explanatory factors identified. Studies are very numerous and have been undertaken over many years. Generally two objectives can be discerned, on the one hand, there is tendency for studies to focus on the performance of peer review as a process that has to meet the needs of various users. These studies tend to offer or to imply recommendations for changes to the way peer review works. On the other hand, a more critical approach can be taken which aims less at offering recommendations for changes to the operation of peer review and more upon observation of and exploration into how peer review functions and how the different actors and aspects of the process relate to each other.

We now consider the various factors which in the literature have been put forward as relating to the fate or outcome for manuscripts submitted to journals. We consider a number of these features and provide a justification for investigating a number of them and why they might relate to citation. We might note that our focus is not upon post-publication peer review—which takes place mostly through so-called mega journals—a process which has become more common (Bjork 2015; Bjork and Hedlund 2015) and is seen by some to have undesirable aspects, see for example work on the journals in the so-called Beall’s List of predatory journals (Shen and Bjork 2015). Our focus here is on the traditional forms of pre-publication peer review as it is within this process that reviewers, editors, and other aspects of the process may have some influence upon the content and citation of the published paper.

It is perhaps helpful to begin this examination of the literature on journal peer review with reference to two relatively recent papers which see the process of peer review as essentially one of choosing good papers and rejecting bad papers. And while it is in part correct that journal peer review seeks to make decisions about what is good and what is not—and indeed ultimately a decision has to be made about whether to include a paper or reject it—the approach exemplified by these writers represents a relatively narrow and abstract view of peer review as a decision making system. The more substantial body of literature which we discuss later and draw on in various ways emphasises the important role of *factors* which affect the judgement of quality made in peer review and the *constructive* role of journal peer review where reviewers and editors contribute to the formation of a text that is eventually published.

The approaches taken by Ma et al. (2013) and Somerville (2016) adopt a system level and abstract perspective and focus on the validity of judgements about inclusion and exclusion of papers. Ma et al. (2013) propose an indicator that combines four other ratio measures to create a composite predictor which they term the EPR, the effectiveness of peer review. The indicator is constructed by using a numerator, comprised of a hit ratio that captures the risks of good papers being accepted, multiplied by a rejection ratio that gives the risk that reflects the number of papers actually rejected compared to the number of papers that should be rejected. The denominator is similarly derived, and is comprised of a leakage ratio multiplied by a miss ratio. The leakage ratio is the number of papers that should be rejected but are accepted compared to the number of papers that are actually accepted. The miss ratio is the number of papers that should be accepted compared with the number actually rejected. The authors calculate their EPR for 28 psychology journals for the years 2008–2010, and which they term an effectiveness of peer review (EPR) indicator. The authors operationalize the indicator by considering papers that should not be accepted but which are and those which are published but uncited, while data on the sizes of the other sets is taken from publishers’ data, which of course includes the number of papers published. The indicator proposed may have some usefulness as a guide; however, the EPR values calculated for each period (annual indicators are given) vary significantly, raising the question of whether the indicator reflects submission rates more than actual peer review effectiveness, and the assumption that papers that are uncited are those which should not have been is another assumption potentially weakening the method adopted.

The approach proposed by Somerville (2016) follows a similar approach in that derives a general model, again based on the definition of sets of papers. It employs concepts of false positive papers (papers accepted by a journal but which should be rejected) and false negatives (papers which are not accepted but which should be published). Somerville’s approach establishes prior probabilities from the acceptance data of the Diamond List in Cabell’s Directory. His model leads to the important conclusion that peer review can have little effect on the quality of papers in a journal when the journal accepts a small number of papers and where the number of submissions is large.

These papers rightly draw attention to the volume of submitted work, i.e. the input of papers, the scope to accept (i.e. to publish papers) and the capacity of the process, in terms of the time and resources to be allocated to reviewing. While this is a useful perspective, and raises the important issue of journal size—in terms of the amount of space journals have to publish papers—the actual internal processes are not investigated. A related work which complements the system level perspective adopted by the authors discussed above is the work on the seasonality of paper submission by Shalvi et al. (2010) who observed that authors that submit papers in the winter months have a better chance of acceptance than if their work is submitted in the summer when journal peer review processes tend to be more overloaded.

We now examine the important work that has looked more closely at features internal to the process of journal peer review, beginning with the factors that affect the judgement of quality. We then move to a discussion of how the peer review process influences the content and quality of the papers submitted. Questions on how content and quality are influenced sit within the context of decisions about judgement, and, as we have indicated above, ultimately journal editors must decide whether to accept a paper or reject it. However, within the process of peer review, the issue of how content and citation are affected is important.

The literature on the topic of judgement in peer review is diverse and very extensive. Much has been made of the subjectivity of peer review, and this has been supported by studies which have shown that peer reviewer’s assessments of the same paper may often disagree. For an earlier by but important work on the sources of bias in peer review see Bakanic et al. (1987), and for a recent large survey of the literature, see Bornmann (2011). Some of the findings on the point of subjectivity raised by these authors and others are very noteworthy. Peters and Ceci (1982) report that in social sciences, previously published papers have a 90% chance of rejection if they are submitted to other journals. These differences in assessment have though been argued to be overstated (Hargens and Herting 1990), and have arisen when papers of very similar quality have been compared.

As the peer review process involves two main types of actor, the editor of the journal (or sub-editor as it is likely to be in the case of a larger journal) and the referees themselves who are normally two in number—but this can vary—various writers have focused upon agreement between the judgements of these two groups. The work of Hargens and Herting (2006) examined this aspect of the peer review process, studying the recommendations of referees and comparing them with the assessments made by editors. While they found substantial agreement within a journal between the combination of reviewers’ recommendations and that of the editor, the degree of agreement varied markedly between journals. The degree of agreement found at the extremes of quality (here between referees and editors) is consistent with their views in the earlier paper (Hargens and Herting 1990) where referee judgements were compared. In a study of papers submitted to their own journal (Cardiovascular Research), Opthof et al. (2002) also note a general level of agreement between reviewers and editors on accepted papers, and also on future citation, but never at a high enough level to remove the need for papers to be sent out to reviewers. Specific reviewer characteristics have been suggested, such as nationality. Opthof et al. (2002, p. 339) state, referring to research by Link (1998): ‘..reviewers from the USA had a preference for manuscripts from the USA compared to reviewers from outside the USA. Such a difference was not seen for manuscripts outside the USA.’

Looking more at the way in which reviewing take place and the interaction between authors, editors and reviewers, there is awareness that double-blinding, signing and other techniques of managing the review process (Godlee et al. 1998; Lock 1994) may encourage confidence and are important aspects in raising submission levels. There has also been discussion of the rejection and submission formats with Range and Tingstrom (1992) arguing for the adoption of the reject and resubmit option in certain cases as a way of giving authors more freedom to decide what to do with their paper, and particularly to avoid long waiting times.

We now consider work on the contribution of the reviewing process to the quality of the paper—the formative evaluation aspect of the peer review process. Here, there is an increasing number of studies, indicating growing interest in peer review and its wider functions and not simply its judgemental/screening purpose (Tennant et al. 2017). We firstly examine a number of earlier, pioneering papers that attempted to investigate what happened inside peer review and sought to establish measures to establish how reviewers contributed to quality.

The study by Goodman et al. (1994) attempted to measure the impact of peer review upon the quality of a paper by using a framework that compared reviewer’s assessments on a numeric scale of the quality of papers on the following six dimensions: Title and Abstract; Introduction; Methods, Results, Discussion and Conclusions and a General Evaluation (Goodman et al. 1994, p. 12). While their study shows an improvement in the quality of manuscripts as identified by their before and after comparison, the differences—across the various dimensions are not large. An important further contribution has also been made to this debate by Bakanic et al. (1987). In examining peer review of manuscripts at the American Sociological Review, the authors took the number of revisions and the number of days between submission and decision (referred to in the literature as the turnaround time) into account in modelling the review process of the journal in which the dependent is an ordinal variable denoting the final outcome. They concluded that the number of revisions and the length of time manuscripts spent in the reviewing process, the greater the likelihood of eventual acceptance. The authors conclude their paper by asserting that ‘peer review plays an important role in the revision, and reconstruction of manuscripts reviewed for publication in ASR’ (p. 640) further observing the absence of other studies on other discipline areas. Their explanation suggests that contrasts between fields and in particular the difference between the physical and biological sciences on the one hand and the social sciences on the other may be explained by differences in the division of labour.

It is our view that the number of revisions and other factors related to the revision of submitted papers might indeed be related to quality, and following Bakanic et al. (1987), we might argue that the need for ‘contribution’ from referees might be greater whenever the number of authors is lower (i.e. the division of labour is less), and that, following their argument, such a variation might be visible to a lesser extent within subfields.

Other important contributions that are more recent also argue for the importance of reviewing as a means of improving quality. Casnici et al. (2017) note the importance of the number of times a paper is reviewed as a possible cause of the higher citation rates of papers. They studied papers which had been rejected by The Journal of Artificial Societies and Social Simulation (JASSS) but which were subsequently published elsewhere. The authors also made the discovery of a possible link between the improvement in the quality of a paper and the extent to which referees of a paper disagreed in their assessments of it. The notion that diversity of views about a paper amongst those reviewing it might be connected with improvements in its quality is given some further weight by the study conducted by Casnici et al. (2017). This study noted that papers reviewed by multi-disciplinary teams were more likely to receive citations than those reviewed by peers from the same discipline.

Thus, while Bakanic et al. (1987) consider that turnaround time reflects the effort given to the improvement of papers, a number of other writers who have examined the duration of the time papers spend in review as relevant to the question of how well academic journals serve their various users [authors and readers (Taylor 2009)], how well knowledge is transferred in critical areas such as medicine where delay in publication might have significant consequences for human health (Chen et al. 2013) and some have made suggestions to improve the process for example Bagla and Mishra (2011). Others have examined the length of time before publication (not necessarily time to acceptance) to determine if journal editors manipulate the queue of publications ready for print in order to boost the journal impact factor (Martin 2016).

Others have considered that the time taken for papers to be reviewed may indicate how valuable an acceptance from a particular journal would be with authors differing in their preparedness to wait for a review decision depending upon their perception of the importance of the journal. This research confirms differences in fields noted above in terms of turnaround time, and that they are more important than career stage of the submitting author, which, while important, is less important than discipline (Poelmans and Rousseau 2015). The work of Bjork and Solomon (2013) does indeed note significant differences between fields noting that within the business and management field “publication delays” lead to on average a duration of 18 months to publication compared with 9 months in the case of chemistry. The length of time spent in revision of papers (the turnaround time) has also been viewed as an undesirable aspect of peer review, in effect a pathological condition of the process of research.

## Methodology

### Initial assumptions

Notwithstanding the range of influences that may affect the number of revisions of a paper and the amount of time in which the paper is in review, there is in our view justification for an attempt to examine the effect of reviewing. As citation data is now increasingly available, it is possible to relate the reviewing effect to one of the most important outcomes of the review after the decision to publish or to reject the paper, and this outcome is the *citation impact of the paper*. Our study is, we believe, possibly the first specifically to make this connection. It does so at the level of a single journal published in the Decision Sciences: Management Science and Operations Research Economics, Econometrics and Finance subject area and which is indexed by Scopus.

Our use of citation *qua* impact of the paper is not a simple equating of citation with quality. Papers may be cited heavily for the errors which they commit and their citation counts measure the visibility in a field and recognition for a variety of purposes. As Leydesdorff (1987) wrote in 1989, citation is certainly multi-valent, a fact which many later authors have explored, one example looking at author motivation is Case and Higgins (2000). We now move to consideration of the statistical methods we have adopted to analyse the evidence we have found.

### Developing a model

There are a number of difficulties in making this analysis at any level, including at the level of the individual journal as we do here. The manuscripts which a journal receives may in many cases already have been rejected by other journals and therefore have been subject to peer review and its effects prior to submission to the journal in question. Furthermore, as Bakanic et al. (1987) note, with some considerable justification, there is no measure of quality or interest of a paper that can be applied to a manuscript which is submitted to a journal, and the only measures that could apply can be found within the peer review process itself or post-peer review in the form of citation counts: ‘no one has devised a measure of manuscript quality independently of publication’(1987, p. 634), and therefore the added value of the peer review process is in principle impossible to infer directly. We do not doubt the possible importance of the effects of prior peer review experience of a manuscript submitted to a journal upon its interest and tendency to be cited later, and we note the lack of a standard that could be applied to submitted papers.

In regard to the first objection to an analysis of this kind, we believe that by using a multivariate method that includes factors/variables that describe the review process and other factors known to affect citation, if aspects of the reviewing process of the journal have no relation to the outcome of the papers in terms of citation impact then the variables that describe the reviewing process will not be significant variables in the model. In respect of this second objection, there is in principle no standard or test of value other than citation that constitutes a reliable assessment of the value of a paper in terms of its interest. The approach we have adopted here is to employ a small sample of papers which, because they are from a single journal, are more likely to have similar editorial control processes applied to them.

We now describe our selection of variables with which to examine the effect of reviewing and the subsequent interest of the paper to future writers—the paper’s impact. The reviewing and editorial process can be described in a number of ways. We believe that two measurements of the process are good quantitative measures of aspects of the reviewing process. The first is turnaround time, which is the amount of time the paper spends in review from the point at which it is received for review to the point at which it is accepted. This factor provides a measure of the effort required to move the paper from the point at which it is thought appropriate for publication, but subject to the need for further work, and the point when the paper is accepted.

The second indicator which describes an aspect of the reviewing process—and in particular the amount of extra attention the paper will receive and the extra work done upon it within the reviewing process is the *original decision* made about the paper. In our data set of papers, which contains papers that were eventually accepted, papers are initially judged at first review as being immediate accept, requiring minor revisions, requiring major revisions or being in a revise and resubmit category. As we note later in our results section, we grouped these four categories of papers into two categories for the analysis as this provided two groups of papers of similar size. We discuss our reasons for this in the results section.

As we have noted above, additionally we aim to control for factors that might affect citation. Here we took account of Baldi (1998) whose work indicated the importance of the following in influencing citation positively: the count of authors (a point on which Abramo and D’Angelo (2015) also agree); length of article; and relevance of the article to “recent work” (Baldi 1998, p. 841) in influencing citation. The variable we constructed to describe recent work was a dummy variable coded one if the paper included any one of the top four keywords in use in the set of papers under examination.

We also aim to examine the effect of the number of keywords as papers with a higher number of keywords might again be more difficult to find reviewers for and (once published) have fewer citations. We also consider that number of authors might affect the reviewing process in a number of ways. Following Bakanic et al. (1987), a larger author team might reduce the need for rewriting of a paper as specialization ensures all parts of the paper are completed. This would reduce the turnaround time of the manuscript. The length of the paper was also considered and this is included in our model as longer papers were, we considered, likely to need more reviewing effort. We also ensured that our papers were of one type only as different types of academic paper have different citation characteristics.

The variable we chose to measure citation impact—i.e. the interest of the paper to later writers—needed to allow comparison of papers published at different times. The choices open to us to create a variable that would allow us to analyse the variable for citation impact using multi-variate methods were twofold. Firstly, one approach might have been to take the basic count divided by elapsed time (citations plus 1/time between publication and census date on which the citation counts were measured) and employ a Tobit model that addresses the issue of the left censoring of the citations (as published papers cannot have fewer than zero citations).

The alternative was to use the transformation of the kind employed by other writers (see the discussion in Rigby (2008) on the use of the ArcSinh transformation) and then employ OLS regression in a multivariate model. This form of model building takes the ArcSinh of the average count of citations per year for each paper as the dependent variable. For all papers, 1 was added to the count of citations to ensure the ArcSinh variable had a value other than 0. This variable for citation impact was computed by dividing a paper’s citation count by the time taken between the year the paper was published and the point in time when the citation data were obtained (March 2017). To ensure completeness of our analysis and to rule out biases in either of the models, we used both the Tobit analysis and the ArcSinh transformation with OLS.

### The data set

We obtained from a journal in the Business and Management literature records on the peer review activities of all papers in the journal over a 6 year period. This information was provided on condition of anonymity. Data were provided with the following fields: Internal identifying code, Article title, Authors, Start page, End page, DOI (if available i.e. normally allocated when published), URL, Keywords, Volume, Vol published year, Issue, Scopus citation Link, Scopus citation count. Other data were then obtained from another part of the publisher’s database for each paper: First submission date, First submission year, Original Decision, Final Submission Date, Final Submission Year, Manuscript Type, Manuscript Status, Decision, Days between Original Submission and Original Decision, Total Days in Review from Original Decision to Final Decision, the number of submissions, number of reviewers in addition to editor involved in the review of the paper. This data was inspected and a data file containing the following fields was prepared for review and checking.

1. Content Item ID provided by the journal and its publisher;
2. Article Title;
3. Authors;
4. Keywords;
5. Year Published;
6. Year Submitted;
7. Original Decision Date;
8. Manuscript Type;
9. Decision;
10. Turnaround Time.
11. Number of Submissions;
12. Count of Non Editor Reviews;
13. DOI.

Using the DOI from the publisher’s data, the following fields of data were then cross-checked with Scopus.

1. Number of Authors;
2. Start Page;
3. End Page;
4. Issue;
5. Citation Count.

### Analysis

The initial data set obtained from the publishers records comprised 598 publications shown below in Table 1 which were at different stages of the reviewing process. These papers were chosen to be of the same type in order to enhance the consistency of the analysis. The papers were original articles, the principle format for high quality publication in the journal. Papers of the following types were removed from the analysis, Book Reviews, Conference Reports, and “Retrostrategy”.

**Table 1:** Total of all papers submitted by year in the period 2010–15 and by current editorial decision

Year	Accept	Reject	Major revision	Minor revision	Reject and resubmit	Grand total	Rejection rate (%)
2010	**34**	2	30	18	18	102	2
2011	**41**	4	22	18	11	96	4
2012	**42**	6	41	28	15	132	5
2013	**29**	20	22	16	11	98	20
2014	**30**	18	36	24	6	114	16
2015	**23**	10	6	11	6	56	18
Grand total	**199**	60	157	115	67	598	10

Around 100 papers were submitted for most of the years, although in the last year for which data was collected, only 57 papers were submitted. Of the 598 papers submitted in the period, 199 were accepted over the period 2010–2015, while 60 were rejected. The rejection rate for the whole period is around 10% but this proportion changes from year to year with the rejection rate being very low for the first three years, and then rising for the remaining three years. The column Grand Total is the sum of the papers in the previous five columns. The rejection rate is calculated as the number of papers rejected, divided by the Grand Total. Papers which are in revision (Major or Minor) or which are in the Reject and Submit Category are in this data set still in the process of revision and resubmission and were not included in the analyses of either turnaround time or of the relationship between the reviewing process and ultimate outcome.

The analysis therefore focused upon papers where a formal decision to accept had been reached by the editor of the journal at the point the data was downloaded, which was in March 2017. These papers are noted in the first column of data in the first table. To help identify the set, these annual paper numbers and the grand total are in bold.

The analysis of the link between aspects of the reviewing process and citation was carried out on a small subset of the 199 papers, comprising 152 papers. This smaller set of papers excluded two types of papers from the analysis which we thought might make our analysis unreliable: (a) those submitted in 2015, of which there were 23, as the citation counts of such papers was likely to be very low and possibly unrepresentative; (b) a further 44 papers were excluded from the set of 199 accepted papers as no DOI was logged in the publisher’s data, and no citation count could be considered accurate as there was doubt over the identity of the paper.

## Results

### Descriptive analysis: review process variables

We reviewed the data set for the period 2010–2015 and report the information on the major variables of note, which are those concerned with the reviewing process, and with citation impact.

#### Review process: turnaround times

**Fig. 1:**
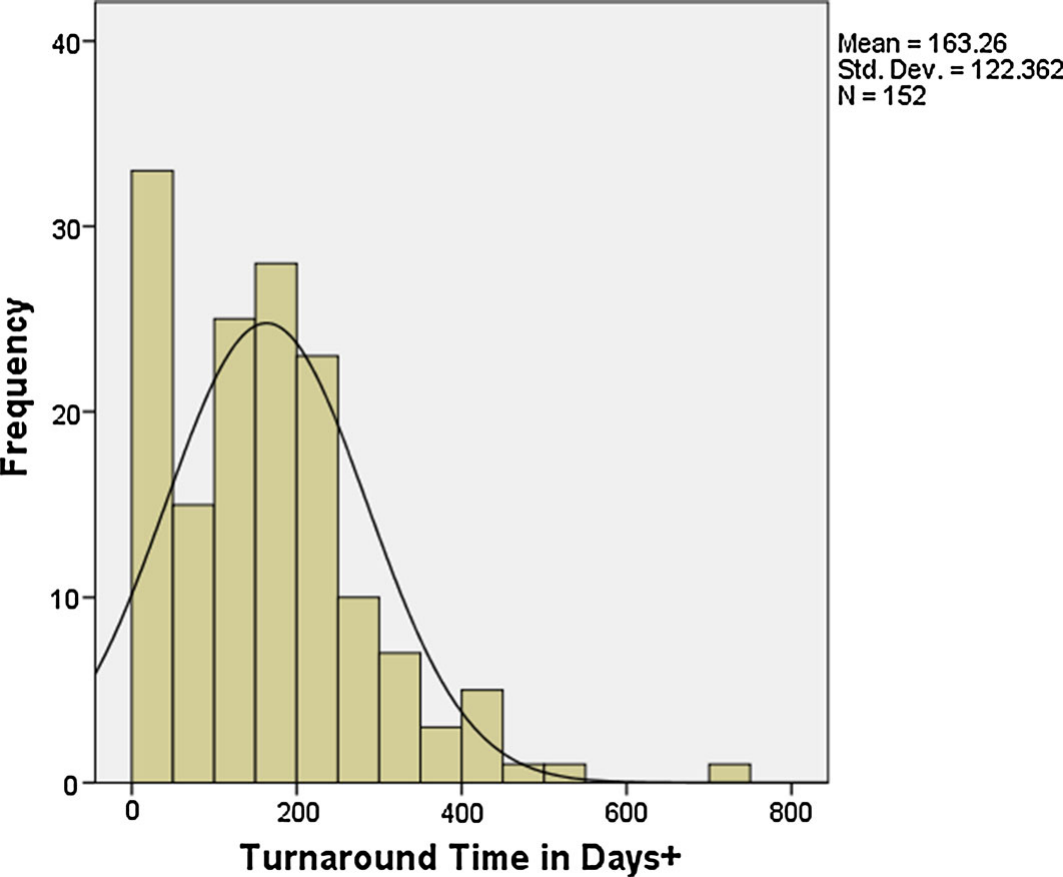
Frequency couny of papers by turnaround time

As can be seen in the following Fig. 1, the distribution of turnaround times would follow a normal distribution were it not for the presence of a group of 18 papers that have very short turnaround time. These are papers that appear to be accepted immediately upon submission or up to around 14 days after submission. Taking all the papers into account including the papers which are immediately accepted, the average waiting time is 5.4 months. If the papers which are accepted in the first 2 weeks are excluded from the set, the average turnaround time is just over 6 months. There are some extreme values at the other end of the distribution. 23 papers have turnaround times of longer than 9 months and 11 papers have turnaround times longer than a year with one paper having a turnaround time of nearly 2 years.

#### Review process: revision effort

Before we examined the effect of the reviewing process on subsequent citation, we were concerned to examine the original decisions made by the editor of the journal to see to what extent these have on the subsequent submission process and need for resubmission. We expected to see some consistency in our data on the question of whether papers that were considered to need more work at first review did indeed receive more work as measured by the number of revisions which they underwent. We investigated whether papers that were subject to a requirement for major revisions, or a revise and resubmit judgement, needed more resubmissions than papers that were in the categories “accept” or “minor revision”. Within our data set there was information concerning the original decision made about papers received on initial submission. There were four categories of initial decision. The first of these was “Accept”, the second “Minor Revision”, the third “Major Revision”, and the third “Reject and Resubmit”. The data from the journal for all papers subsequently accepted (and therefore published) is given below.

The evidence of the frequency counts from table suggests that the initial “original decision” about the paper and its need for further work is not a random event and that there is an association between the original decision on the need for re-work of the paper and what subsequently happens in terms of revisions.

Analysis of the data in Table 2 shows the number of revisions per paper for the papers in the two categories “Accept” and “Minor Revision” is 1.7 while the number of revisions for papers that are in the second categories where the original decision of the editor is that the papers require more work (“Major Revision” and “Reject and Resubmit”) is 2.6. This number is calculated by dividing the number of revisions of all papers by the number of papers which were reviewed. Our evidence does therefore suggest that the original decision of the editor was related to how much further work a paper required. A Chi square test which cross-tabulates the papers by the two categories (type of original decision and by the count submissions) confirmed the statistical significance of the difference with the value of the statistic being 17.0456 and with one degree of freedom the *p* value is 0.000.

**Table 2:** Original decision * count of submissions cross tabulation

		Count of Submissions					Total
		1	2	3	4	5	
Original decision	Accept	39	4	1	0	0	44
	Minor revision	0	31	11	0	0	42
	Major revision	0	34	19	6	2	61
	Revise and resubmit	0	3	2	0	0	5
Total	39	72	33	6	2	152	

Two interpretations of this statistical association may be considered. On the one hand, the initial judgement may be considered to be one of quality assessment where papers that have more interest and better execution are deemed likely to need less amendment and subsequently, because the changes required are less onerous, fewer resubmissions are required. On the other hand, this link between initial decision and the number of subsequent revisions can be seen to be a simple matter of cause and effect without any reference to the quality of the paper as such: papers requiring more work are given an initial assessment that more revisions are required, and, in the course of amending the manuscript, this greater level of work needs more resubmissions to revise the paper to meet the original criteria for acceptance. Our test is not able to distinguish between these different explanations, but our view is that the data is an indicator of the reviewing effort. We therefore created a variable “Revision Effort” by combining the accept and minor revision papers into one set of low effort papers and the major revision and revise and resubmit papers into another set of high effort papers.

### Results of applying the model of reviewing and effect on citation

We present two models of the effect of reviewing on citation impact. One model uses an OLS regression with the dependent variable being the ArcSinh transformation of the citation impact. The other model applies a Tobit regression. The following tables provide the results of the statistical tests. Tables 3, 4 and 5 relate to the regression using the ArcSinh transformation, while Table 6 provides the information from the Tobit regression. Table 3 shows the results of the model, Table 4 gives the results of the ANOVA table and Table 5 gives the coefficients (unstandardized and standardized) and significance levels of the predictor variables.

**Table 3:** ArcSinh model summary

Model summary									
Model	*R*	*R* square	Adjusted *R* square	Std. error of the estimate	Change statistics		df1	df2	Sig. *F* change
					*R* square change	*F* change			
1	0.412^a^	0.169	0.135	0.495514	0.169	4.930	6	145	0.000

^a^Predictors: (Constant), Review_Effort, Count of authors + , Article length + , Key_Topic_Dummy_4 + , Count of keywords + , Turnaround time in days +

**Table 4:** ArcSinh model ANOVA

ANOVA^a^						
Model		Sum of squares	df	Mean square	*F*	Sig.
1	Regression	7.262	6	1.210	4.930	0.000^b^
	Residual	35.602	145	0.246		
	Total	42.865	151			

^a^Dependent variable: ArcSinh_Citation_Count

^b^Predictors: (Constant), Review_Effort, Count of authors + , Article length + , Key_Topic_Dummy_4 + , Count of keywords + , Turnaround time in days +

**Table 5:** ArcSinh model coefficients

Coefficients^a^						
Model		Unstandardized coefficients		Standardized coefficients	*t*	Sig.
		*B*	SE	*β*		
1	(Constant)	1.096	0.205		5.345	0.000
	Review_Effort	0.327	0.094	0.305	3.463	0.001
	Turnaround time in days +	− 0.001	0.000	− 0.236	− 2.627	0.010
	Count of authors +	− 0.036	0.029	− 0.097	− 1.275	0.204
	Count of keywords +	− 0.056	0.024	− 0.185	− 2.354	0.020
	Article length +	0.000	0.001	0.023	0.297	0.767
	Key_Topic_Dummy_4 +	0.195	0.086	0.183	2.267	0.025

^a^Dependent variable: Arcsinh_Citation_Count

**Table 6:** Tobit regression

Tobit regression					Number of observations 152		
LR					chi^2^(6) = 27.09		
Prob >					chi^2^ = 0.0001		
Log likelihood = − 269.469					Pseudo *R*^2^ = 0.0479		
Citation_Rate_Inc_Self_Citation_	Coef.		SE	*t*	*P* > *t*	[95% CI]	
Review effort	1.221		0.334	3.660	0.000	0.562	1.881
Turnaround_Time_Days	− 0.004		0.001	− 2.530	0.013	− 0.006	− 0.001
Count_of_Authors	− 0.112		0.099	− 1.130	0.259	− 0.307	0.083
Count_of_Keywords	− 0.205		0.085	− 2.410	0.017	− 0.374	− 0.037
Article_Length	0.000		0.005	− 0.050	0.963	− 0.009	0.009
Key_Topic_Dummy_4	0.658		0.303	2.170	0.031	0.060	1.257
_Cons	1.034		0.720	1.430	0.153	− 0.390	2.457
/Sigma	1.7039	0.1094	1.487	1.9203			
Obs. summary							

25 left-censored observations at at Citation_Rate_Inc_Self_Citation_< = 0

127 uncensored observations

0 right censored observations

Both models suggest that the reviewing process is related to citation impact. In both models the extent to which a paper is reviewed is related to its citation impact; however, the turnaround time is also significant in the models but it is negatively associated with citation impact. Thus, the longer a paper is in review, the more likely it is to have lower citation rate, all other relevant factors considered.

Of the other variables that we considered could affect the citation impact of papers (count of authors, length of the paper, count of keywords and the presence of key topic keywords), it is the count of keywords and the presence of key topic keywords which are positively associated with citation impact of the paper. While neither model has a high predictive power, both models do perform similarly in that in each, the same variables affect the dependent variable, citation impact.

## Discussion

### Limitations of the study

We must first note a number of caveats about our work: these are mainly limitations and qualifications of our findings that relate to the general context in which we have carried out the work, but there are some specific points about the analysis.

Initially, we should note that our study is significantly context bound and is therefore likely to be of limited applicability to other fields, and we do not make the claim that our findings are widely applicable. The area in which we have carried out our research, which is the field of management and business, is very different from other areas of scientific enquiry. As Bjork and Solomon (2013) note in their study of publication times (which includes turnaround time) and editorial practice, differences between fields are significant with, at the one end, the Business and Management field that has a total publication delay of just under 18 months (in their survey), which is twice that of the field with the shortest publication delay, Chemistry. As regards editorial practice and author behaviour within the management and business field, studies such as that by Ellison (2002) on the field of economics do indeed suggest that papers have multiple revisions and that this number has grown over time. Certainly, there is a consensus that in the context our work is done, there is a far greater chance of multiple revisions and journal peer review takes longer than in scientific disciplines such as medicine.

We should also note that our analysis has not considered all the papers that have been received by the journal in the period studied. Clearly, the information we were seeking to use for our analysis concerned citation counts of papers and we therefore excluded papers that were still in the reviewing system, some of which are likely to have been in review for a considerable period. This therefore limits our description of the *system of peer reviewing* in the journal we have chosen. But this is in fact an unavoidable constraint arising from the need to consider papers which had citations (and been accepted). Consequently, papers which are still in review, of which there were a number in the journal, were excluded from the analysis.

We believe however, that empirical studies, even if they are limited in scope as ours has been, are valuable and have the potential to enlarge the debate about peer review, a debate which will only benefit from the engagement of more of the key actors (authors, editors, publishers and reviewers) and the availability, and analysis, of more evidence.

### Findings and reflection

Our analysis has demonstrated that, in the limited case we have chosen of the behaviour of reviewers, editors and authors in a single journal, and over a relatively short period in the history of that journal, there is a positive effect of our variable for reviewer effort—the count of reviews—upon the citation impact of a paper. By citation impact we mean the recognition and visibility that a paper achieves. We do not equate citation simply with quality. We also note a negative effect between the length of time a paper has spent in review and the citation impact, all other things considered. So it is not the amount of time a paper spends in review (the turnaround time) which matters to its subsequent citation but the engagement with reviewers that has an effect. We also note positive effects of the count of keywords, and the presence within the paper of discussion of key topics—as evidenced by the presence of relevant keywords.

The effect of the count of reviews—the intensity and effort expended on reviewing a paper—on the subsequent citation impact suggests the credibility of the view emerging in the literature that peer reviewing has a constructive and not merely judgemental function in relation to the papers submitted.

Let us briefly consider though, a sceptical line of argument which could be made against the case we have outlined. That sceptical line of argument is that peer review is simple error removal. If the reviewing process was essentially a judgemental activity by which errors are corrected (with papers that are highly erroneous excluded altogether—although we have no data on this aspect in our study), the reviewing effort variable would either have no statistical relationship with the outcome variable or, and this is more likely, it would have a negative relationship in that papers that needed more work at first review (a higher reviewer input) would have less recognition and general interest ultimately as such papers contained more errors to begin with. The analysis of our data, however, shows a positive connection between the effort of reviewing and the outcome variable. Under the assumption that peer review is merely error checking, this association would be a paradox which one could state thus: manuscripts at review that were thought to be in more need of correction were, at publication found to be of greater interest.

As we have argued above, the knowledge production interpretation of the relationship appears more likely than the error correction interpretation. In this interpretation, our variable of review effort is a measure of the input to the manuscript from the reviewing process far more than we consider it to be an assessment of the extent of citation, and we have little evidence to draw the conclusion that the reviewing process misjudges the quality of manuscripts. Rather, as we have said above, we favour the interpretation based on our construction of the reviewing effort variable that it is the reviewing effort applied to the paper (by authors, editor and referees) that may in some way be responsible for an enhancement of the attractiveness and interest of manuscripts.

How exactly peer reviewing performs as a knowledge production process, and is distinct from authorial creativity and knowledge generation, is clearly, on the basis of our study open to question. The literature suggests a number of dimensions to which we believe our evidence may point. Firstly, and perhaps closest in form to judgmental peer review itself is a dimension concerned with the removal of errors in the submitted paper’s text that subsequently strengthens the argument, and thereby leads to higher levels of citation. Other literature cited above suggests that variety, in terms of the number of reviewers and in terms of the disciplines of the reviewers involved, may play a role in manuscript development. While increasing the number of reviewers of different types increases the chance that errors will be picked up, we consider that diversity is likely to add conceptual dimensions to the submitted work, either developing concepts or arguments already present in the work or introducing new and complementary or even contrasting ones.

We consider that there might well be three main dimensions to the improvement of the paper. Firstly, as we have suggested above, increasing review may reduce the number of errors present in the text. Secondly, an increasing number of reviews may help to realize the quality already inherent in the paper with the reviewing and resubmission process being nothing more than a mechanism for the realization of the manuscripts’ latent qualities. Thirdly, increasing review might add substantively to the manuscript with the enhancement in the recognition of the paper coming from the addition of new concepts, and their elaboration and incorporation.

If now we return briefly to the issue of how long papers are in review before they are published (the turnaround time), we note that in our analysis, the amount of time papers spend in review is negatively associated with the citation impact of the paper. So while the number of times papers are revised (review effort) appears to lead to higher recognition for the paper (a higher citation count), a variable that might be thought to be positively linked to the number of revisions of a paper is negatively associated with the paper’s level of recognition. Our explanation of this relationship is that the time taken—we have referred to this as turnaround time—is better seen as a delay that may result from difficulties that the submitted work presents to the reviewing process. The delay might be caused by problems identifying suitable reviewers, or when authors have difficulty in dealing with the comments of reviewers. Our conclusion is therefore that time in review, contrary to our original assumption, is not a measure of or indicator of knowledge generation.

### Implications

In the literature review we discussed the differences in peer reviewing practices that prevailed between different subject fields. We might speculate that in fields where papers have fewer resubmissions (and the quantity of rework is less), the effect of revising the paper upon citation will be less. A suggestion for further research is to examine the reviewing practice in other journals and other fields where different writing and contribution practices apply.

We have not established that peer review is a knowledge production process *sui generis*, but our work suggests that some form of knowledge production is taking place and we have suggested a simple typology. We suggest that reviewers should be credited with the contribution they make and that authors of papers be invited more formally to outline how they believe their work has benefited from peer review. Our suggestions tend towards what is already practice in open peer-review and post-publication peer review contexts.
